# Association of State Boards of Dental Examiners

**Published:** 1884-09

**Authors:** 


### Association of State Boards of Dental Examiners.

SARATOGA, Aug. 8th, 1884.

The Association was called to order by President Taft. The States represented were: New Jersey by F. C. Barlow, Fred. A. Levy, C. W. Maloney; Vermont by Jos. Lewis, G. H. Swift, S. B. Hodge; West Virginia by C. A. Wingerter ; Maryland by

E. P Keech; Pennsylvania by C. N. Pierce, J. C. Green, E. T. Darby, and S. H. Guilford; Ohio by J. Taft and H. A. Smith; Illinois by A. W. Harlan and G. A. Cushing; Georgia by G. W. McElhaney.

Dr. Taft said that a committee was appointed by the College Faculties Association to confer with this body. A motion was carried to invite them in. Dr. Abbott was the only member of that Association present. Dr. Abbott stated the formation of the Association of College Faculties, as elsewhere reported in the REGISTER. He then read the resolutions passed by that body. The action of the Supreme Court in Illinois gives this Association of Boards a great deal of power over the standing of the graduates of the colleges, and then a little harmonious work on the part of these two bodies may induce all colleges to join the Association, the result being an elevation of the standard. It was moved that the resolutions be accepted. Carried. A letter was read from M. H. Chappell, Secretary of the Indiana State Dental Association, of which the following is an extract:

"The Board of Examiners of Indiana have used their utmost endeavors to have the Indiana Dental College alter their *advertised* standard and conform to the National Board instructions. Whether it was the admonitions from the Illinois Board or from the Indiana Board, we can say that the trustees have *positively decided* to conform, and they now appeal to the Indiana Board to ask of the National Board to remove any injunction that may appear against said college. Henceforth the Indiana Dental College will join with the Indiana Board in the work of the National Board."

No action was taken on the letter further than to accept and put it on file. Dues were then paid; adjourned to 3½ P. M. At this hour President Taft called the meeting to order, and Secretary Cushing read the minutes of the former session. Dr. G. W. McElhaney, of Georgia, requested his State put on the list with the others, as he was not present in the morning. Dr. Keech announced that the dental law in Maryland had just been passed, and mentioned two cases of men who had been practicing dentistry in part at the time the law was passed, one a mechanical dentist, the other simply an extractor and applier of leeches, and the Doctor wanted to hear from the menbers as to the custom in treating such cases. An Auditing Committee, consisting of Drs. Pierce, McElhaney, and Lewis, was appointed to audit bills and vouchers. Dr. Guilford offered a resolution to the effect that the proceedings of both meetings of this Association, together with the report of the Association of College Faculties, be published in pamphlet form and that a copy be sent to each college and State Board. The resolution was adopted. Dr. Cushing offered the following resolution: *Resolved*, That this Association instructs its members after June, 1885, to recognize as reputable no dental college that does not require, as a requisite for graduation, attendance upon two full regular courses of lectures and practical instruction, which courses shall be of not less than five months duration and shall be held in separate years, with practical instruction intervening between the courses. Such colleges must require a preliminary examination before admitting students to matriculation. Resolution carried unanimously. Dr. McElhaney offered this resolution: *Resolved*, That this Association recommends the dental colleges not to grant free scholarships to State societies through the recommendation of the Examining Boards or of State societies. On motion the resolution was adopted. Dr. Pierce offered the following: *Resolved*, That this Association insists that no Board, hereafter organized and connected with this body, shall confer degrees or titles of any nature. Dr. Guilford moved to amend by rescinding resolution three on page seven of the published proceedings. Carried. Dr. Pierce again offered his resolution, which was carried. Prof. J. Taft was elected President unanimously; Dr. McElhaney was elected Vice-President; Dr. Cushing was elected Secretary and Treasurer. The Association agreed to meet in New Orleans the last Tuesday in March, 1885.

Adjourned.

---

To Disguise the Taste of Iodide of Potash.—M. Gerard Lague (*Lyon Med.*) states that syrup of gooseberry will completely disguise the taste of iodide of potash.

